# Body weight, body composition and energy balance related behaviour during the transition to parenthood: study protocol of a multi-centre observational follow-up study (TRANSPARENTS)

**DOI:** 10.1186/s12889-019-6884-0

**Published:** 2019-05-06

**Authors:** Tom Deliens, Vickà Versele, Hannelore Vanden Eynde, Peter Clarys, Roland Devlieger, Annick Bogaerts, Leonardo Gucciardo, Annick Schreurs, Caroline Van Holsbeke, Dirk Aerenhouts

**Affiliations:** 1 Faculty of Physical Education and Physiotherapy, Department of Movement and Sport Sciences, Vrije Universiteit Brussel, Pleinlaan 2, 1050 Brussels, Belgium; 2 Faculty of Medicine, Department of Development and Regeneration, KU Leuven, Herestraat 49, 3000 Leuven, Belgium; 3 Obstetrics and Gynaecology, University Hospitals KU Leuven, Herestraat 49, 3000 Leuven, Belgium; 4 Faculty of Medicine and Health Sciences, Centre for Research and Innovation in Care (CRIC), University of Antwerp, Universiteitsplein 1, 2610 Wilrijk, Belgium; 5 Faculty of Medicine, Department of Obstetrics and Prenatal Medicine, Vrije Universiteit Brussel, Laarbeeklaan 101, 1090 Brussels, Belgium; 6 Obstetrics and Prenatal Medicine, University Hospital Brussel, Laarbeeklaan 101, 1090 Brussels, Belgium; 7 Obstetrics and Gynaecology, Jessa Ziekenhuis, Stadsomvaart 11, 3500 Hasselt, Belgium; 8 Obstetrics and Gynaecology, Ziekenhuis Oost-Limburg, Schiepse Bos 6, 3600 Genk, Belgium

**Keywords:** Pregnancy, Parenthood, Body composition, Energy balance related behaviour

## Abstract

**Background:**

The transition to parenthood is a cornerstone event for both parents, potentially leading to relevant changes in lifestyle and behaviour. In women, the metabolic changes during and after pregnancy and the deleterious effects of excessive gestational weight gain and postpartum weight retention have been extensively described. However, there is no full understanding about which specific energy balance related behaviours (EBRB) contribute to unfavourable weight gain and weight retention. Furthermore, information on how transition to parenthood affects men is lacking. Therefore, this study aims to investigate changes in body weight, body composition and EBRB in couples transitioning to parenthood.

**Methods:**

TRANSPARENTS is a multi-centre observational follow-up study that focuses on body weight, body composition and EBRB during the transition to parenthood. Couples (women and men) will be recruited during the first trimester of their first pregnancy. Study visits will occur at four occasions (12 weeks of pregnancy, 6 weeks postpartum, 6 months postpartum and 12 months postpartum). Anthropometrics of the parents and new-borns will be assessed including body weight, height/length, body composition (using bio-electrical impedance analysis and measurement of four skinfold thicknesses (biceps, triceps, subscapular and supraspinal/suprailiac)) and waist and hip circumference. Dietary intake, physical activity, sedentary behaviour, smoking habits, sleeping pattern, fatigue, diet and exercise related partner support, mental health, breastfeeding, contraception use, and socio-demographics will be assessed using a questionnaire. In addition, accelerometry will be used to assess physical activity and sedentary behaviour objectively. Also data from women’s medical record, such as pre-pregnancy weight and pregnancy outcomes, will be included. Multilevel modelling will be used to evaluate maternal and paternal changes in body weight, body composition and EBRB during and after pregnancy (primary outcomes). Multiple linear regression analyses will be performed to identify predictors of changes in body weight, body composition and EBRB. All analyses will be adjusted for possible confounders.

**Discussion:**

TRANSPARENTS is a unique project identifying vulnerable parents and (un)favourable changes in EBRB throughout this potentially critical life period. Provided insights will facilitate the development of effective intervention strategies to help couples towards a healthy transition to parenthood.

**Trial registration:**

Clinicaltrials.gov Identifier: NCT03454958. Registered March 2018.

## Background

The worldwide prevalence of overweight and obesity has increased epidemically during the last decennia [1–3], with the greatest prevalence being observed in upper-middle-income level regions such as North America and Western Europe [4, 5]. In Belgium, about half of the adult population is overweight or obese [6], and prevalence of maternal obesity in Europe reaches 25% [7]. Predictions show that these trends of weight gain are to be continued throughout the next couple of decades [8, 9]. Overweight and obesity are major risk factors for non-communicable diseases such as cardiovascular diseases, type 2 diabetes, musculoskeletal disorders, and a number of cancers, and are estimated to account for 3.4 million deaths per year globally [3, 4, 10, 11]. Next to increased mortality rates, co-morbidities attributable to overweight and obesity cause decreased quality of life as well as a large and still growing economic burden [11, 12]. This highlights the urgency to develop effective strategies for the prevention of overweight and obesity.

Although overweight and obesity are caused by a multitude of factors (i.e., genetic, metabolic), the rapid increase in prevalence suggests that, rather than biological changes, behavioural and environmental influences predominate [13]. Weight gain is typically caused by an energy imbalance between energy consumed and energy expended [3]. It is recognised that this imbalance is due to alterations in energy balance related behaviour (EBRB), namely poor eating behaviour characterized by increased intake of energy-dense foods high in fats and free sugars, decreased physical activity levels, and increasing sedentary lifestyles [3].

It has been argued that specific transitional phases during the lifespan, such as living together, marriage, or pregnancy/having a child may have a strong effect on people’s long-term EBRB and body weight [14]. Identification and thorough understanding of critical periods when people are more likely to gain weight or display unhealthy changes in EBRB could help us to develop more effective intervention programs, focusing on that particular stage of life. In this study we will focus on the transition to parenthood, i.e. having a first child.

Research showed that pregnant women are at substantial risk for excessive gestational and postpartum weight gain and retention [15]. Excessive weight gain during pregnancy increases the risk for gestational diabetes, pregnancy-induced hypertension, large-for-gestational age infants etc. [16–19], whereas higher postpartum weight retention can increase risks in a consecutive pregnancy [20] and lead to lifelong obesity and its related non-communicable diseases [21]. A US study demonstrated that parents gain weight more rapidly than childless women and men [22]. A recent Belgian study showed a mean postnatal weight retention of 3.3 kg, with women retaining up to 16.2 kg of weight six weeks after delivery [23]. Evidence further suggests that increased gestational weight gain (GWG) and postpartum weight retention is more likely for the first pregnancy [20, 24]. A recent review also revealed that pregnancy-related weight gain coheres with increases in fat mass in US women [25].

Increased postpartum body weight is generally caused by a combination of both retention of GWG and alterations in lifestyle due to increased demands related to time, finances, fatigue and child care responsibilities [24, 26, 27]. Healthful EBRBs are associated with reduced risk for excessive GWG [28], and thus, in turn, with reduced risk for excessive postpartum weight retention. Recent reviews [29, 30] emphasize the importance of regular physical activity regarding health during preconception, pregnancy and postpartum. However, parenthood and physical activity are often inversely related [31]. A large prospective study in Australia [32] showed that moderate-to-vigorous physical activity declines during the transition to parenthood in both women and men. Similar results were found in a prospective Canadian study reporting decreases in moderate-to-vigorous physical activity but also in sedentary time, while an increase in light intensity activities was observed [33]. Regarding eating behaviour, most studies seem to focus on pre- and early pregnancy nutrition and its effect on infant outcomes [34]. Although the transition to parenthood may negatively impact healthful eating, studies investigating changes in eating behaviour prospectively are scarce. An Australian study reported no significant changes in diet quality and takeaway-food consumption among women and men having their first child [35]. It should be noted, however, that caloric intake was not studied in this study, so it is not clear whether energy balance was disrupted through excess energy intake. In addition, screening of the literature has taught us that (change in) sedentary behaviour remains understudied during this critical life phase. Although there are some Australian, Canadian and US prospective studies on distinct behaviours, to the best of our knowledge, there are no studies investigating pregnancy-related weight gain from a holistic energy balance perspective, i.e. considering eating, physical activity and sedentary behaviour simultaneously. Besides, Western European research on this topic is lacking. Considering the fact that EBRB is strongly culturally related (e.g. differences in eating or physical activity culture between the US, Australia and Europe), it is important to study changes in body weight, body composition and EBRB along with their determinants among child expecting couples in Western Europe.

Most studies investigating changes in body composition or EBRB during the transition to parenthood focus on women while neglecting their life partners. A recent review [36] revealed that fathers spend less time on moderate-to-vigorous physical activity compared to childless men, especially when they have young children. The same review [36] called for further longitudinal research investigating in detail how the arrival of a child influences physical activity behaviour among fathers. Our review of the available literature revealed that prospective studies investigating body weight and composition, and eating and sedentary behaviour among men transitioning to fatherhood are lacking. Moreover, most weight gain/retention interventions focus on the mother, whereas interventions focusing on the couple while making use of mutual reinforcement, may be more efficacious [33]. Also, if both parents display certain behaviours from early on during the upbringing of their child, it is more likely that children will display similar behaviours during childhood and later life through the principles of parental norms and modelling [37]. Hence, pregnancy should be seen as a window of opportunity to shape healthy EBRB by using a family-centred care approach.

Therefore, the first objective of this study is to investigate both maternal and paternal changes in body weight, body composition and EBRB during the transition to parenthood (i.e. having their first child). A second objective is to investigate socio-demographic and behavioural predictors of changes in both maternal and paternal body weight, body composition and EBRB.

## Methods

### Study setting

TRANSPARENTS is a multi-centre follow-up study in which four Belgian hospitals are involved: University Hospital of Brussels (UZ Brussel), University Hospital of Leuven (UZ Leuven), Jessa Hospital in Hasselt and Hospital Oost-Limburg in Genk. Every year, around 8650 children are born across the four hospitals, of which approximately 45% are firstborn children. Recruitment as well as baseline measurements will take place in the hospitals. Follow-up measurements will take place in the hospitals or at the participants’ homes, depending on their preferences. The protocol of the study and all related documents were reviewed and approved by the Ethics Committees of all the participating hospitals. Written informed consent will be provided by all participants upon entry in the study.

### Recruitment of participants and eligibility criteria

Two researchers will be responsible for recruitment of the participants at the obstetric units of the participating hospitals during the intake visit (around the 8th – 12th week of pregnancy). Each researcher will be assigned two hospitals and will be responsible for the recruitment, baseline measurements and follow-up measurements of the participants from the respective hospitals. To optimise recruitment, posters and flyers will be placed in the waiting rooms of the participating hospitals, encouraging potential participants to visit the study website. The flyers will also be handed over by the nurses/midwives of each obstetric unit to couples expecting their first child, and they will be encouraged to fill in a contact sheet when the researcher is not available for informing potential participants. Couples who left their contact details will be contacted by phone or email, depending on their preference, to provide information and ask them to take part in the study.

Only couples expecting their first child (nulliparous women and childless men) can participate in the study. Because of anthropometric and energy balance related differences between men and women (e.g. different dietary habits, differences in physical activity pattern, etc.) [38], we decided not to include lesbian or homosexual couples, as this would result in a heterogenous “partner group” (i.e. consisting of both men and women). Therefore, only male-female couples and only couples of which both parties are eligible and consent to participate will be included in this study. Dutch speaking participants aged 18 year or older, with any body mass index (BMI) and any socio-economic status can participate. Exclusion criteria for participants are the following: unable or unwilling to give informed consent; suffering from a pathological condition that may influence EBRB (e.g. diabetes), having a significant psychiatric disorder, a recent history of bariatric surgery, pregnant through in vitro fertilisation treatment or with requirements for complex medical diets; unable or not allowed to exercise because of medical or other specific reasons (e.g. bed-rest); having a multiple pregnancy (twin, triplet, etc.).

### Sample size

For the main outcomes of this study – changes in body weight, body composition and EBRB – a relatively small effect size of 0.15 is anticipated, as this was the minimum effect size observed in a previous study in which changes in body weight and EBRB during another critical life period (transition from secondary to tertiary education) were investigated [39].

With a statistical power of 0.8, significance level (α) of 0.05, and taking into account an estimated maximum attrition rate of 75%, a minimum of 248 participants (= 124 couples) will be recruited. The number of participants to be recruited in each participating obstetrics unit will depend on the number of new-borns in the respective hospitals. Taking into account the relative yearly share of new-borns in each hospital, minimum targets for UZ Brussel, UZ Leuven, Jessa Hospital and Hospital Oost-Limburg are set at 34, 35, 26 and 29 couples respectively. The expected duration to reach the total target of 124 couples is four months.

### Timing

**Fig. 1:**
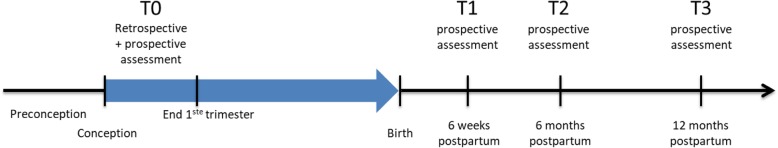
Timeline of the TRANSPARENTS observational follow-up study

Couples will be recruited around week 8–12 of pregnancy. Baseline measurements will take place during the twelfth week of pregnancy. Follow-up measurements will take place at six weeks, six months and one year after delivery (Fig. 1). A range of two to three weeks before or after the theoretic measurement point will be allowed. Baseline measurements will take place in the hospitals before or after the routine pregnancy ultrasound evaluation at week 12 of gestation or during a home visit if this is not possible. Follow-up measurements will take place in the hospitals or at the participants’ homes, depending on their preferences.

### Data collection

#### Medical record data

Pre-pregnancy weight will be collected from the electronic medical record at inclusion (T0). Complications during pregnancy (e.g. pregnancy diabetes, pre-eclampsia, hypertension), GWG, method of delivery (spontaneous/vaginal, cesarean section) and new-born birthweight will be collected from the electronic medical record after the delivery.

#### Anthropometrics

Anthropometrics of both parents will be assessed at all four time points (T0-T3) following guidelines of the International Society for the Advancement in Kinanthropometry (ISAK) [40]. Height will be measured using a calibrated SECA wall mounted stadiometer (1 mm accurate scale). Body weight (0.1 kg accurate scale) and body composition (fat mass, fat free mass, muscle mass, bone mass, extra-cellular water, intra-cellular water and organ fat) will be measured using TANITA MC780SMA Bio Impedance Analyzer (BIA). BIA is an inexpensive, rapid, non-invasive and safe method for estimating body composition [25, 41]. BMI will be calculated through dividing body weight (in kg) by squared height (in m). A Harpenden skinfold caliper (0.2 mm accurate scale) will be used to measure skin fold thickness at the biceps, triceps, subscapular and supraspinal sites. Cescorf measuring tape (1 mm accurate scale) will be used to measure waist (at the narrowest part of the waist) and hip circumference (at the largest part of the hip), in order to estimate abdominal and gluteofemoral body fat, respectively. Also new-borns’ anthropometrics will be objectively assessed by the researcher. Therefore, body weight, length, head and upper arm circumferences, and biceps, triceps, subscapular and suprailiac skin folds of the new-born will be measured at T1, T2 and T3. All data will be collected consistently by the same two qualified researchers. Researchers will be trained by a certified ISAK anthropometrist in order to collect data in a standardised way [40]. The training will consist of a theoretical and practical part in which the measurements (incl. landmarks) will be explained and demonstrated. In this training session, the researchers will perform several repeated measurements on the abovementioned body sites on several test subjects under supervision of the certified ISAK trainer. To guarantee reliability between the researchers, a second practical session will be organised to further optimize measurements and minimize inter-rater measurement error.

#### Questionnaires

Questionnaires will be used to collect prospective data across all time points including self-reported body weight and height and EBRB, consisting of weight control behaviour, dietary intake (22-items Food Frequency Questionnaire (FFQ) [42]), physical activity (IPAQ – Dutch version) [43] and context-specific sedentary behaviour (adapted from Busschaert et al. with pregnancy and child-birth related sedentary behaviours such as ‘sitting while breastfeeding’ [44]). Besides these EBRB related questions, the questionnaire will also include other health related factors (smoking habits, sleeping pattern, fatigue), questions to assess social support from the partner for eating and physical activity behaviour (based on the Social support for diet and Social support for exercise questionnaire [45]; adjusted for partner support and translated (independently by two different researchers and afterwards put together) to Dutch), psychological wellbeing (Edinburgh Postnatal Depression Scale (EPDS) and Gotland Male Depression Scale (GMDS)), breastfeeding and contraception use (T1-T3), medical conditions and socio-demographics (age, ethnicity, level of education, employment and marital status). The baseline questionnaire (T0) will include additional retrospective questions about pre-pregnancy body weight, changes in EBRB since pregnancy, smoking habits, sleeping pattern and fatigue since pregnancy and nausea during pregnancy (Pregnancy-Unique Quantification of Emesis/Nausea (PUQE)). A link to the questionnaire will be sent by email to each participant (men and women separately). Participants who do not complete the questionnaires within one week will be reminded up to three times by email or text message.

#### Accelerometry

Tri-axial accelerometers (Actigraph GT3X+) will be used to measure physical activity and sedentary behaviour objectively over a one-week period. The accelerometers will be programmed on a sampling frequency of 30 Hz and downloaded at an epoch of 60 s. Data processing will be done with Actilife software. Time spent sedentary and in light, moderate and vigorous physical activity will be calculated in minutes using Freedson cut-off points [46]. Accelerometers will be handed over to the participants on the day of the anthropometric assessments. Starting from the next day, participants will be asked to wear the devices from waking up until going to bed for seven consecutive days. Participants will be asked to remove the accelerometer while swimming, showering or bathing, as well as to keep an activity log writing down such non-wearing intervals. The accelerometers should be returned per pre-franked post package, which the participants receive together with the accelerometers. A detailed overview of the above measurements, methods and time points is presented in Table 1.

**Table 1:** Overview of measurements and used methods

Measurements	Measurement tool (number of measures per occasion)	T0		T1	T2	T3
		Retrosp.	Prosp.			
Anthropometrics (parents)						
Body weight	TANITA Scale (once)		X	X	X	X
Body height	SECA Stadiometer (twice)		X	X	X	X
Self-reported weight and height	Questionnaire	X	X	X	X	X
Body composition	TANITA Bio-electrical Impedance Analyzer (once)		X	X	X	X
Skin folds (biceps, triceps, subscapular and supraspinal)	Harpenden skinfold caliper (twice)		X	X	X	X
Waist circumference	Cescorf measuring tape (twice)		X	X	X	X
Hip circumference	Cescorf measuring tape (twice)		X	X	X	X
Anthropometrics (new-born)						
Body weight	SECA Baby Scale			X	X	X
Length	SECA Measuring board (twice)			X	X	X
Head and upper arm circumferences	Cescorf measuring tape (twice)			X	X	X
Skin folds (biceps, triceps, subscapular and suprailiac)	Harpenden skinfold caliper (twice)			X	X	X
EBRB and other health related factors						
Weight control behaviour	Questionnaire	X	X	X	X	X
Changes in dietary and physical activity behaviour since pregnancy	Questionnaire	X				
Dietary intake	Questionnaire: 22-item FFQ		X	X	X	X
Self-reported physical activity	Questionnaire: IPAQ		X	X	X	X
Context-specific sedentary behaviour	Questionnaire		X	X	X	X
Objective physical activity and sedentary behaviour (7 days)	Accelerometry (Actigraph)		X	X	X	X
Smoking habits	Questionnaire	X	X	X	X	X
Sleeping pattern and fatigue	Questionnaire	X	X	X	X	X
Psychosocial						
Partner support for diet and exercise behaviour	Questionnaire		X	X	X	X
Psychological wellbeing	Questionnaire: EPDS and GMDS		X	X	X	X
Socio-demographics and other sample characteristics						
Breastfeeding and contraception use	Questionnaire			X	X	X
Medical conditions (high blood pressure, high cholesterol, heart and vascular diseases, cancer, sport injuries, depression, chronical fatigue, insomnia)	Questionnaire		X	X	X	X
Emesis/Nausea	Questionnaire: PUQE	X				
Socio-demographics (e.g. age, ethnicity, level of education, employment and marital status)	Questionnaire		X	X	X	X
Pre-pregnancy weight, fertility treatment	Medical Record	X				
Complications during pregnancy (e.g. pregnancy diabetes, pre-eclampsia, hypertension), pregnancy weight gain, way of delivery (natural, cesarean section) and new-born birthweight	Medical Record			X		

*IPAQ* International Physical Activity Questionnaire, *FFQ* Food Frequency Questionnaire, *EPDS* Edinburgh Postnatal Depression Scale, *GMDP* Gotland Male Depression Scale, *PUQE* Pregnancy-Unique Quantification of Emesis/Nausea

### Outcomes

The primary aim of the study is to evaluate maternal and paternal changes in body weight, body composition and EBRB during and after pregnancy up to one year after delivery. Therefore, the primary outcomes are changes in body weight, BMI, body composition (fat mass, fat free mass, muscle mass, bone mass, extra-cellular water, intra-cellular water and organ fat), dietary intake, physical activity and sedentary behaviour. Secondary outcomes are socio-demographics, breastfeeding, partner support for diet and exercise behaviour, psychological wellbeing and new-borns’ anthropometrics.

### Data management

An electronic software program (RedCap software) will be used to enter and store data from the medical records, anthropometrics, and questionnaires.

### Statistical analysis

All statistical analyses will be performed in SPSS 25.0 and R (RStudio version 1.1.383). Prior to the statistical analyses, all data will be screened for outliers and data cleaning will be performed when necessary. Descriptive statistics of the sample will be calculated and drop-out analysis will be conducted, using independent samples *t*-tests for quantitative data and *chi*^*2*^ for qualitative data. Multilevel modelling will be performed to evaluate changes in body weight, body composition and EBRB over time according to gender. Multilevel modelling is particularly well fitted to analyse repeated measures over time in a hierarchical structure (i.e. individuals clustered within couples and clustered within hospitals) including missing values (which are anticipated to be present due to the wide range of variables at different time points we aim to measure) [47]. Multiple linear regression analyses will be performed to identify predictors of changes in body weight, body composition and EBRB. All analyses will be adjusted for possible confounders. *P*-values < 0.05 will be considered as statistically significant.

## Discussion

The transition to parenthood is hypothesized to be a critical period in life where changes in body weight and EBRB are likely to occur. Unhealthy changes in EBRB, excessive weight gain during pregnancy, and weight retention after delivery may lead to long-term weight retention. In turn, this may lead to increased prevalence of overweight and obesity, which are major risk factors for non-communicable diseases. Moreover, women showing excessive weight gain during pregnancy are at increased risk for pregnancy- and child-related complications [16–19, 48]. Hence, it is important to unravel these mechanisms causing excessive weight gain throughout this life event, which may all interrelate and have an impact on weight gain and retention during the transition to parenthood. TRANSPARENTS is an innovative study investigating changes in body weight, body composition and EBRB during the transition to parenthood from a holistic point of view. Moreover this research will not only focus on women, but also on their male partners. The combination of investigating changes in body weight and body composition, in association with changes in EBRB, of both women and men transitioning to parenthood will provide understanding about this life phase and those most at risk for excessive weight retention. This knowledge will help us to develop family-based interventions targeting excessive weight gain and unfavourable changes in EBRB among couples having their first child. As lifestyle interventions focusing only on health behaviour generally show moderate effects, adding the aspect of partner support might be an important asset for extra encouragement to change towards, and even more importantly, maintain healthy behaviours [49, 50].

A first strength of this study is the longitudinal follow-up design, since research on EBRB before, during or after pregnancy is mainly based on cross-sectional designs [31]. A second strength is the abovementioned holistic approach of EBRB (i.e. combining eating, physical activity and sedentary behaviour) in relation to changes in body weight and body composition. Furthermore, also partner support and psychological health are taken into account. Third, the majority of studies on this topic have focused on women while leaving men ignored [31]. Hence, studies including both women and men transitioning to parenthood are needed to enhance the knowledge on this critical period in life. Fourth, the use of objective measurements of anthropometrics, physical activity and sedentary behaviour can be considered an important strength as they increase accuracy and reduce measurement error compared to subjective measurements. Unfortunately, no objective measurements, that can be used on a large scale, exist to assess dietary intake. Finally, the multi-centre approach (i.e. recruitment at four participating hospitals across two provinces in Flanders and the Brussels Capital Region (Belgium)) can be considered an important strength of this study.

A first limitation of this study is the retrospective collection of pre-pregnancy data (as of course, it is impossible to know when people are getting pregnant). Second, because of feasibility reasons we chose not to include a control group (couples not expecting a child), as we would need matched couples who are in the same life phase (except for pregnancy) and do not have children nor a wish for children during the upcoming study period. Finally, due to the recruitment of volunteers we may expect a selection bias, as studies investigating health behaviour generally end up with a healthier, but also higher educated study sample compared to the general population [51].

The TRANSPARENTS study is the first European study investigating changes in EBRB during and after pregnancy up to one year after delivery, and its role in excessive GWG and postpartum weight retention taking into account both women and men. Provided insights from the TRANSPARENTS study will facilitate the development of effective intervention strategies to counter excessive weight gain and retention among couples transitioning to parenthood.

## Abbreviations

BMI: Body mass index
EBRB: Energy balance related behaviour
EPDS: Edinburgh Postnatal Depression Scale
FFQ: Food frequency questionnaire
GMDS: Gotland Male Depression Scale
GWG: Gestational weight gain
IPAQ: International Physical Activity Questionnaire
PUQE: Pregnancy-Unique Quantification of Emesis/Nausea
